# Granulomatöse rosazeaartiges Exanthem unter Tofacitinib

**DOI:** 10.1007/s00105-021-04793-6

**Published:** 2021-03-24

**Authors:** Kristina Neumann, Patrick Terheyden, Diamant Thaçi

**Affiliations:** 1 Exzellenzzentrum Entzündungsmedizin, Universitätsklinikum Schleswig-Holstein – Campus Lübeck, Ratzeburger Allee 160, 23538 Lübeck, Deutschland; 2 Klinik für Dermatologie, Allergologie und Venerologie, Universitätsklinikum Schleswig-Holstein – Campus Lübeck, Ratzeburger Allee 160, 23538 Lübeck, Deutschland

**Keywords:** Januskinase-Inhibitoren, Colitis ulcerosa, Dermatitis, Acne vulgaris, Nebenwirkung, Janus kinase inhibitors, Colitis, ulcerative, Dermatitis, Acne vulgaris, Side-effect

## Abstract

Wir berichten von einer 32-jährigen Patientin mit Colitis ulcerosa, die unter Therapie mit dem JAK(Januskinase)-Inhibitor Tofacitinib eine massive papulopustulöse Dermatitis entwickelt hat. Trotz intensiver lokaltherapeutischer Maßnahmen und der Einnahme von Kortikosteroiden und Doxycyclin trat keine ausreichende Besserung ein, sodass die Tofacitinib-Behandlung beendet werden musste. Bekanntermaßen kann die Klasse der JAK-Inhibitoren zu infektiösen und allergischen Hautnebenwirkungen führen. Fälle von steriler papulopustulöse Dermatitis unter Therapie mit JAK-Inhibitoren sind allerdings bislang kaum berichtet worden.

## Abstract (en)

The case of a 32-year-old female with ulcerative colitis who developed severe papulopustular dermatitis while undergoing treatment with the Janus kinase (JAK) inhibitor tofacitinib. Despite intensive topical therapy, treatment with oral corticosteroids and oral doxycycline was unable to achieve sufficient improvement. Hence, tofacitinib treatment needed to be discontinued. It is well known that the class of JAK inhibitors can cause infectious and allergic cutaneous side effects. However, sterile papulopustular dermatitis as a side-effect has rarely been reported to date.

## Falldarstellung

### Anamnese

**Figure 1:**
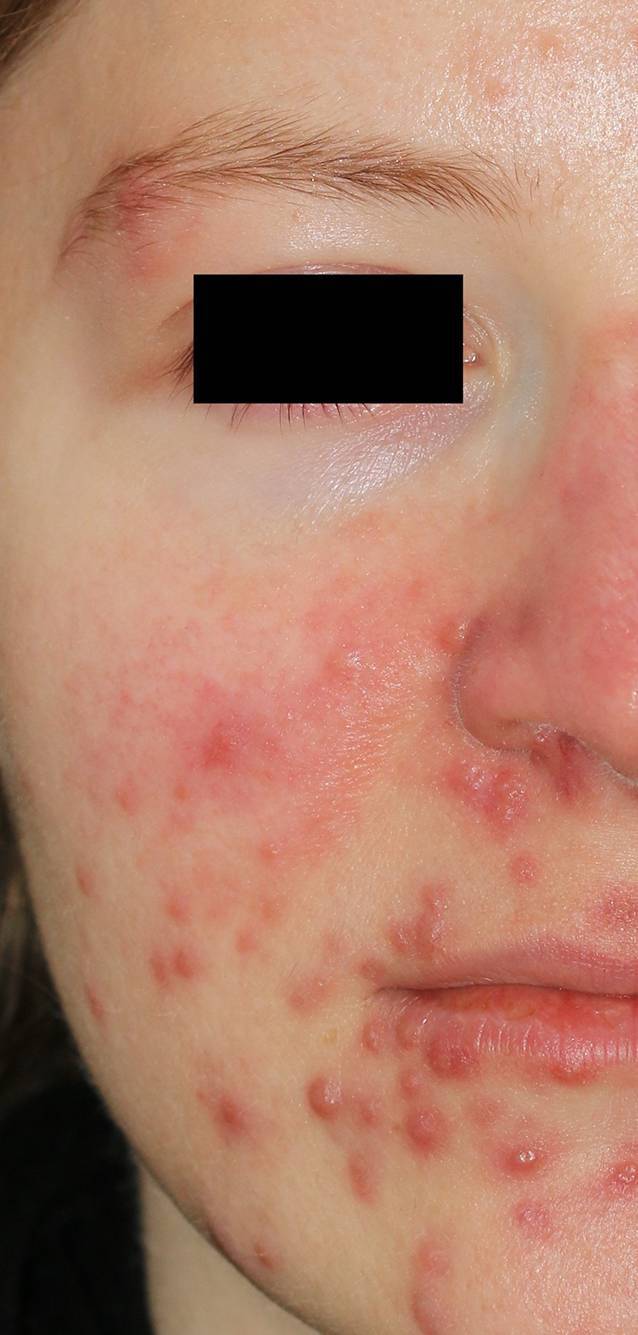

Die 32-jährige Patientin berichtete, dass sie aufgrund einer Colitis ulcerosa seit 9 Wochen den JAK(Januskinase)-Inhibitor Tofacitinib (2-mal täglich 5 mg) einnehme. Die Diagnose Colitis ulcerosa war in der hiesigen Gastroenterologie bei passender klinischer Symptomatik und wiederholten segmentalen Darmbiopsien mit histologischer Aufarbeitung gestellt worden. Seit 3 Wochen bestanden massive papulopustulöse Hautveränderungen im Gesicht (Abb. 1).

Zuvor sei die Patientin immer hautgesund gewesen. Insbesondere die Anamnese bezüglich Acne vulgaris und Rosazea war unauffällig.

### Klinischer Befund

Bei Vorstellung in unserer Klinik zeigten sich massive papulopustulöse Hautveränderungen im Gesicht. Komedonen lagen nicht vor (Abb. 1).

### Diagnose

**Figure 2:**
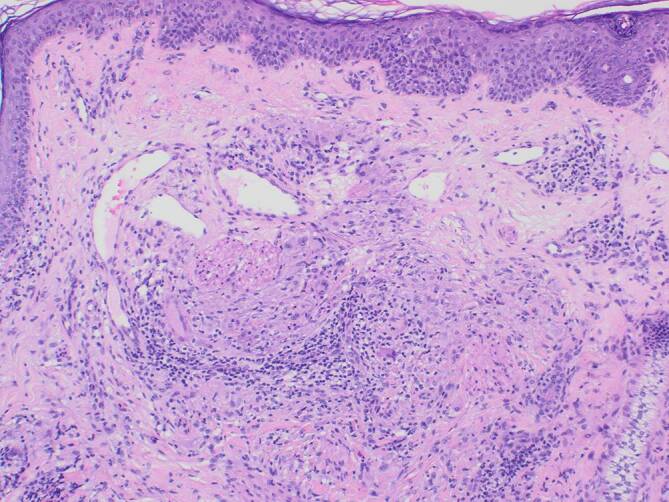

Der Hautabstrich einer Pustel zeigte physiologische Hautflora. Eine läsionale Probebiopsie war mit einer granulomatösen Rosazea vereinbar. Histopathologisch zeigte sich kein Nachweis von Hefen oder Milben (Abb. 2).

### Therapie und Verlauf

Eine orale Therapie mit Doxycyclin in einer Dosierung von 100 mg täglich sowie eine Lokaltherapie mit fusidinsäurehaltiger Creme wurden eingeleitet. Die Befundkontrolle nach 10 Tagen zeigte jedoch keine Besserung. Aufgrund des massiven Befundes mit hohem Leidensdruck der Patientin erfolgte die Hospitalisierung. In Rücksprache mit den behandelnden Gastroenterologen wurde die Behandlung mit Tofacitinib abgesetzt. Wir führten die orale Antibiose mit Doxycyclin fort und ergänzten eine orale Prednisolontherapie mit 1 mg/kg/KG einmal täglich über 3 Tage. Lokal wurde mit metronidazolhaltiger Creme und Pimecrolimuscreme behandelt. Unter der Therapie kam es zur raschen Besserung des Befundes. Die Therapie der Colitis wurde bei bereits gebesserten Hautbefund auf Ustekinumab (IL[Interleukin]-12/23-Inhibitor) umgestellt.

Eine klinische Verlaufskontrolle 4 Wochen nach Entlassung zeigte einen erheblich gebesserten Befund.

## Diskussion

Die Gruppe der JAK-Inhibitoren umfasst eine Gruppe von Medikamenten, die für unterschiedliche Indikationen zugelassen sind oder sich im Rahmen klinischer Studien in Erprobung befinden. Man unterscheidet 4 Subtypen von Januskinasen: JAK1, JAK2, JAK3 und Tyk2 (Tyrosinkinase 2; [1]). JAK-Inhibitoren blockieren den intrazellulären JAK-STAT(„signal transducer and activator of transcription“)-Signalweg und weisen durch die Transkription von Zielgenen eine entzündungshemmende, immunmodulierende oder immunsupprimierende Wirkung auf. Aktuell sind für unterschiedliche Indikationen Tofacitinib, Baricitinib, Upadacitinib und Ruxolitinib zugelassen. Tofacitinib ist in Kombination mit Methotrexat für die Psoriasisarthritis und die rheumatoide Arthritis zugelassen und als Monotherapie für die Colitis ulcerosa. Bekannte Nebenwirkungen umfassen neben Infektionen Blutbildveränderungen mit Anämie, Lymphozytopenien und Neutropenien, Hyperlipidämien, Leberwerterhöhungen und Virusreaktivierungen [2]. Als Nebenwirkungen an der Haut sind bakterielle Infektionen gut bekannt. Auch Exantheme im Sinne allergischer Reaktionen von Sofort- und Spättyp treten auf [2].

Bei unserer Patientin lag bei Nachweis steriler Pusteln keine eitrige Follikulitis vor. Auch eine Verschlechterung einer vorbestehenden Rosacea papulopustulosa konnte wir aufgrund der zuvor unauffälligen Anamnese, des plötzlichen Auftretens der Hautveränderungen wenige Wochen nach Beginn der Tofacitinibtherapie sowie der Abheilung nach Therapieumstellung weitestgehend ausschließen. Eine sterile papulopustulöse Dermatitis, wie sie bei unserer Patientin auftrat, ist unter Tofacitinib bislang nicht beschrieben. Allerdings kam es in einer rezent publizierten Phase-II-Studie zu einem oralen Tyk2-Inhibitor in 9 % der Fälle zu akneähnlicher Dermatitis [3]. Im Gegensatz zu infektiösen Hautnebenwirkungen, die sich durch die immunsupprimierende Wirkung der JAK-Inhibitoren erklären lassen, ist die Ursache steriler, akne-/rosazeaähnlicher Hautnebenwirkungen bislang ungeklärt.

Obwohl es derzeit kontrovers diskutiert wird, könnte eine Zunahme der Seborrhö und eine Veränderung des Hormonprofils während der Behandlung mit JAK-Inhibitoren mit Auftreten von akneiformen Hautnebenwirkungen verbunden sein. Paradoxerweise wird sogar postuliert, dass bei Rosazea eine Inhibierung des JAK2-STAT3-Signalwegs und bei Akne die Hemmung von JAK1- und JAK3-Aktivität potenzielle Therapieansatzpunkte sein könnten [4].

Sterile akneiforme Hautnebenwirkungen sind bei zahlreichen, unterschiedlichen Medikamenten bekannt. So kommt es bei etwa 44 % der mit Rituximab behandelten Patienten zu kutanen Nebenwirkungen mit Erythemen, Urtikaria und akneiformen Exanthemen [5]. Unter den EGFR(„epidermal growth factor receptor“)-Inhibitoren kommt es bei bis zu 18 % der Patienten zu papulopustulösen Exanthemen. Diese treten, ähnlich wie bei unserer Patientin, oft schon in den ersten Wochen der Behandlung auf. Typischerweise sind papulopustulöse Exantheme unter EGFR-Inhibitoren transient, auf die Dauer der Behandlung beschränkt und heilen nach Absetzen der Therapie meist folgenlos ab. Auch hier sind schwere Verläufe mit hoher Einschränkung der Lebensqualität, die eine spezifische Therapie oder ein Absetzten der Therapie erforderlich machen, beschrieben [6]. Auch unter der Einnahme von MEK(„mitogen-activated protein kinase kinase“)-Inhibitoren treten sehr häufig papulopustulöse Exantheme auf. So kommt es bei 77 % der mit Trametinib behandelten Patienten zu einer zu einer akneiformen Dermatitis [7].

Empfehlungen zur Behandlung dieser kutanen Nebenwirkungen können sich aufgrund der fehlenden Evidenzlage aktuell nur auf die klinischen Ähnlichkeiten mit Akne vulgaris und Rosazea stützen. Neben topischen Vitamin-A-Säure- und tetrazyklinhaltigen Präparaten finden intern auch kurzfristig Kortikosteroide sowie orale Tetrazykline Anwendung [6]. Von einer internen Retinoidtherapie wurde bei unserer Patientin abgesehen, da diese bei Colitis ulcerosa kontraindiziert ist. Im Falle unserer Patientin war aufgrund der Schwere und Hartnäckigkeit des Befundes eine Therapiefortführung nicht zu rechtfertigen, sodass nach Ausschöpfung therapeutischer Maßnahmen die Umstellung auf Ustekinumab erfolgte.

Aktuell stehen für sämtliche Zulassungsbereiche von Tofacitinib, wie rheumatoide Arthritis, Psoriasisarthritis und Colitis ulcerosa, weitere Substanzklassen zur Verfügung, sodass ein Therapiewechsel bei intolerablen kutanen Nebenwirkungen oftmals möglich sein sollte.

## Fazit für die Praxis

- Eine bislang wenig beschriebene Nebenwirkung unter Therapie mit JAK(Januskinase)-Inhibitoren ist das Auftreten steriler, papulopustulöser Exantheme.
- Die Therapie erfolgt analog zur Therapie der Akne vulgaris und Rosacea papulopustulosa.
- Bei unzureichender Besserung stehen in den jeweiligen Indikationen oftmals Ausweichpräparate anderer Substanzklassen zur Verfügung.
